# Kinetic and catalytic mechanisms of the methionine-derived glucosinolate biosynthesis enzyme methylthioalkylmalate synthase

**DOI:** 10.1016/j.jbc.2024.107814

**Published:** 2024-09-23

**Authors:** Vivian Kitainda, Joseph M. Jez

**Affiliations:** Department of Biology, Washington University in St Louis, St Louis, Missouri, USA

**Keywords:** plant biochemistry, glucosinolate biosynthesis, reaction mechanism, Brassicaceae, enzyme kinetics

## Abstract

In Brassica plants, methionine-derived aliphatic glucosinolates are chemically diverse natural products that serve as plant defense compounds, as well as molecules with dietary health-promoting effects. During their biosynthesis, methylthioalkylmalate synthase (MAMS) catalyzes the elongation reaction of the aliphatic chain. The MAMS-catalyzed condensation of 4-methylthio-2-oxobutanoic acid and acetyl-CoA generates a 2-malate derivative that either enters the pathway for the synthesis of C_3_-glucosinolates or undergoes additional extension reactions, which lead to C_4_- to C_9_-glucosinolates. Recent determination of the x-ray crystal structure of MAMS from *Brassica juncea* (Indian mustard) provided insight on the molecular evolution of MAMS, especially substrate specificity changes, from the leucine biosynthesis enzyme α-isopropylmalate synthase but left details of the reaction mechanism unanswered. Here we use the *B. juncea* MAMS2A (BjMAMS2A) isoform to analyze the kinetic and catalytic mechanisms of this enzyme. Initial velocity studies indicate that MAMS follows an ordered bi bi kinetic mechanism, which based on the x-ray crystal structure, involves binding of 4-methylthio-2-oxobutanoic acid followed by acetyl-CoA. Examination of the pH-dependence of *k*_cat_ and *k*_cat_/*K*_m_ are consistent with acid/base catalysis. Site-directed mutagenesis of three residues originally proposed to function in the reaction mechanism—Arg89 (R89A, R89K, R89Q), Glu227 (E227A, E227D, E227Q), and His388 (H388A, H388N, H388Q, H388D, and H388E)—showed that only two mutants (E227Q and H388N) retained activity. Based on available structural and biochemical data, a revised reaction mechanism for MAMS-catalyzed elongation of methionine-derived aliphatic glucosinolates is proposed, which is likely also conserved in α-isopropylmalate synthase from leucine biosynthesis in plants and microbes.

Plants have evolved specialized metabolic pathways through gene duplication and functional divergence of enzymes involved in primary metabolism. Glucosinolates are a diverse class of plant natural products resulting from such a process, namely the gene duplication of enzymes involved in the leucine biosynthesis pathway in plants (1). Glucosinolates are largely found within the members of the family Brassicaceae, which includes vegetables such as *Brassica oleracea* (broccoli, cabbage, kale), *Brassica napus* (oilseed rape or canola), *Brassica juncea* (brown or Indian mustard), and the model plant *Arabidopsis thaliana* (thale cress) (2). Glucosinolate function stems from their chemically and structurally varied hydrolysis products (Fig. 1*A*), which are responsible for the strong flavors of mustard and other Brassica vegetables, as well as serving as plant defense molecules that repel insects, fight fungal infections, and discourage herbivory (3, 4, 5, 6). Certain hydrolysis products, such as isothiocyanates, can potentially serve as cancer prevention agents in humans. For example, in broccoli, 4-methylsulfinylbutyl glucosinolate hydrolyzes to the isothiocyanate sulforaphane, which may prevent tumor growth by blocking the cell cycle and promoting apoptosis (7, 8, 9, 10).

**Figure 1:**
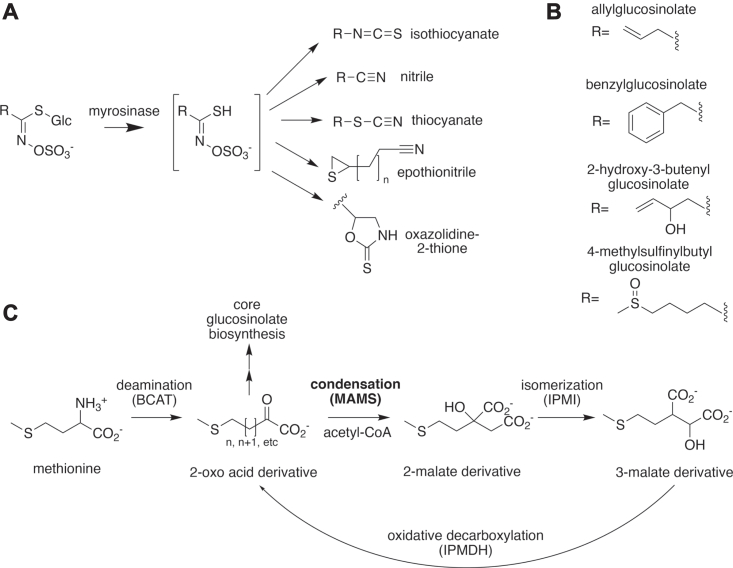
**Glucosinolate structure and biosynthesis.***A*, diversity of glucosinolate chemical structure. In Brassicaceae, wounding or cellular damage mixes glucosinolates and myrosinase, which hydrolyzes the sugar group to produce chemically active compounds with varied structures depending on the glucosinolate R-group. *B*, glucosinolate R-group diversity. Chemical variation is introduced based on the amino acid precursor used in glucosinolate biosynthesis. For example, the 4-methylsulfinylbutyl glucosinolates are derived from methionine. *C*, methionine-derived aliphatic glucosinolate biosynthesis. Elongation of the aliphatic side-chain of methionine involves a series of deamination (branched-chain amino transferase; BCAT), condensation (methylthioalkylmalate synthase; MAMS), isomerization (isopropylmalate isomerase; IPMI), and oxidative decarboxylation (isopropylmalate dehydrogenase; IPMDH) reactions. The resulting 2-oxo acid form can either enter core glucosinolate biosynthesis by a BCAT-catalyzed transamination reaction with the resulting amino acid form as substrates of cytochrome P450 CYP79F (represented by *double vertical arrows*) or undergo additional rounds of the elongation cycle.

The breadth of glucosinolate function results from their varied structures, which comes from the use of aliphatic (alanine, leucine, isoleucine, valine, and methionine), aromatic (phenylalanine and tyrosine), and indole (tryptophan) amino acids as precursors, as well as elongation of some aliphatic side-chains, such as those derived from methionine, by up to nine carbons (1, 3, 11). After formation of the core glucosinolate structure (*i.e.*, a β-D-glucopyranose residue linked by a sulfur atom to a (Z)-N-hydroximinosulfate ester; Fig. 1*A*), the variable side-chains can undergo further chemical modifications (Fig. 1*B*) (3). There are over 130 described glucosinolate structures, of which aliphatic methionine-derived glucosinolates are the most abundant form (3, 11). Although both elongation and chemical modification of amino acid side-chains are important for methionine-derived glucosinolate diversity, the aliphatic chain elongation process (Fig. 1*C*) has not been well described mechanistically, especially considering its importance in efforts to engineer glucosinolate production in plants for plant defense, human health, and nutrition. These efforts are important for combating worldwide crop loss by pathogens, which reduce yields of crops by an estimated 40% and an annual loss of ∼$220 billion in revenue (12).

Although the steps required for methionine-derived glucosinolate biosynthesis are known (Fig. 1*C*), there are varied levels of molecular understanding of the enzymes involved (13). There have been limited studies addressing the first (*i.e.*, deamination of methionine into a 2-oxoacid) and last (*i.e.*, amination of the elongated 2-oxoacid) steps of the methionine-derived glucosinolate elongation pathway. Both steps are catalyzed by branched-chain aminotransferases (BCAT) with one of the seven known BCAT genes in *Arabidopsis* (BCAT4) having a definitive role in glucosinolate biosynthesis; the level of involvement of other BCATs is unclear (11, 13, 14, 15). The second elongation step—condensation of the 2-oxoacid with acetyl-CoA to form a 2-malate derivative—is catalyzed by methylthioalkylmalate synthase (MAMS) isoforms with each isoform allowing a certain number of condensation iterations (Fig. 1*C*) (16, 17, 18). Although MAMS has long been functionally characterized, the x-ray crystal structure of the enzyme from *B. juncea* was only recently solved and biochemical studies of the enzyme have yet to be carried out (19). The third and fourth elongation steps involve isomerization of the 2-malate derivative to a 3-malate derivative by isopropylmalate isomerase (20, 21, 22, 23) and the oxidative decarboxylation of the latter to the elongated 2-oxoacid catalyzed by isopropylmalate dehydrogenase (23, 24, 25, 26). Central to the formation of the abundant methionine-derived aliphatic glucosinolates are the MAMS-catalyzed iterative chain-elongation reactions.

MAMS is evolutionarily derived from α-isopropylmalate synthase (IPMS), which catalyzes the first reaction of leucine biosynthesis, namely the condensation of α-ketoisovalerate (KIV; also known as 3-methyl-2-oxobutanoid acid) and acetyl-Coenzyme-A (acetyl-CoA) (1, 17, 18, 19). Although IPMS is limited to one condensation reaction, in contrast to the multiple condensations catalyzed by MAMS, both enzymes condense a 2-oxo acid substrate with acetyl-CoA, which places IPMS and MAMS in the Claisen-like condensation subgroup of the DRE-TIM metallolyase superfamily (27, 28). In the Brassicaceae, evolution of MAMS from IPMS involved the loss of the C-terminal regulatory domain, which releases the enzyme from feedback inhibition by leucine, and two key amino acid changes in the active site (18, 19). Glycine substitutions for a serine and a proline increase the size of the substrate-binding site to fit the longer side-chains of 2-oxo acids like 4-methylthio-2-oxobutanoic acid (4-MTOB) in MAMS compared to the smaller KIV substrate in IPMS. Different MAMS isoforms synthesize aliphatic glucosinolates of varied lengths. For example, *B. juncea* MAMS1 isoforms generate C_3_- to C_5_-glucosinolates and MAMS2 isoforms perform a single elongation to form a C_3_-glucosinolate (19). The x-ray crystal structure of *B. juncea* MAMS1A in a dead-end complex with CoA and the substrate 4-MTOB provided insight on the sequence changes leading to isoform reaction differences.

The MAMS x-ray crystal structure also led to a proposed chemical mechanism for the condensation of acetyl-CoA with 2-oxo acid substrates, like 4-MTOB, in methionine-derived glucosinolate biosynthesis (19). In the reaction mechanism, a divalent metal, such as Mn^2+^ or Mg^2+^, is essential for activity and interacts with the substrate. A general base (proposed to be His388 in BjMAMS1A) facilitates carbanion formation for the condensation step. Nucleophilic attack of the resulting carbanion on the C2-carbonyl of the 2-oxo acid substrate with the help of a potential general acid (proposed to be Arg89 in BjMAMS1A) leads to condensation of the acetyl-group with 4-MTOB. A water activated by an unidentified general base in the active site likely serves as a nucleophile to react with the thioester carbonyl. The resulting tetrahedral intermediate collapses with the release of CoA and product from the active site.

Here we use BjMAMS2A, which catalyzes the single condensation of 4-MTOB and acetyl-CoA in C_3_-glucosinolate biosynthesis (19), to test the proposed kinetic and chemical reaction mechanisms using initial velocity studies and site-directed mutagenesis. The resulting biochemical data, along with structural comparisons, allowed for the proposal of a modified chemical reaction mechanism for MAMS.

## Results and discussion

### Initial velocity studies and ordered kinetic mechanism of BjMAMS2A

As reported previously (19), different MAMS isoforms from *B. juncea* and *A. thaliana* have similar steady-state kinetic parameters for 4-MTOB and acetyl-CoA. BjMAMS2A was selected for the present studies because it only catalyzes one round of condensation, which simplifies the enzyme assays. The protein was expressed in *Escherichia coli* as an N-terminally His_6_-tagged protein and purified by nickel-affinity and size-exclusion chromatography, as described (19).

**Figure 2:**
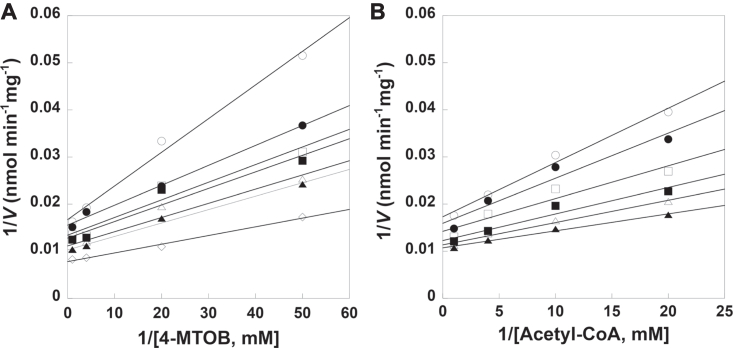
I**nitial velocity analysis of the kinetic mechanism of BjMAMS2A.** Experimental data are indicated by symbols in the double-reciprocal plots of the substrate variation experiments. The lines represent the global fit of all data to the equation for an ordered sequential bi bi kinetic mechanism. Assays were performed as described in Experimental procedures with each data point shown as the average for n = 3. *A*, double-reciprocal plot for 1/*V versus* 1/[4-MTOB, 0.02–1 mM] at 0.1, 0.15, 0.25, 0.35, 0.5, 1.0 and 3.0 mM acetyl-CoA (*top* to *bottom*). *B*, double-reciprocal plot for 1/*V versus* 1/[acetyl-CoA, 0.05–1 mM] at 0.1, 0.125, 0.15, 0.20, 0.25, and 0.5 mM acetyl-CoA (*top* to *bottom*).

Initial velocity data generated by co-varying acetyl-CoA and 4-MTOB were used to distinguish between possible bi bi kinetic mechanisms (Fig. 2). The intersecting initial velocity pattern eliminates a ping-pong mechanism, which also does not agree with the reaction chemistry and x-ray crystal structure of BjMAMS1A (19). Global fitting analysis of the data to equations describing random and ordered sequential kinetic mechanisms yielded a better fit for an ordered mechanism compared to a random one (ordered: r^2^ = 0.958 *versus* random: r^2^ = 0.907), as well as steady-state kinetic parameters (Table 1) comparable with those previously reported for BjMAMS2A (19). Further analysis of the initial velocity data using a secondary plot yielded two linear lines for 4-MTOB and acetyl-CoA, respectively, with different slopes (Fig. S1), which is consistent with an ordered sequential bi-bi kinetic mechanism (29). The sequential ordered bi bi kinetic mechanism observed for BjMAMS2A is similar to the kinetic mechanism reported for the evolutionarily-related bacterial IPMS (30). Both enzymes also share similar active site structures in which the 2-oxo acid substrate (4-MTOB in MAMS and KIV in IPMS) are internally bound and blocked from solvent by binding of acetyl-CoA in the catalytic cycle (19, 31), which results in an ordered substrate addition in the kinetic mechanism.

**Table 1:** Kinetic constants for an ordered sequential bi bi kinetic mechanism of BjMAMS2A

Kinetic constants	Fitted parameters
*V*_max_ (mmol min^−1^ mg protein^−1^)	122 ± 3
*K*_m_^4-MTOB^ (μM)	24.6 ± 0.4
*K*_m_^acetyl-CoA^ (μM)	40.0 ± 1.0


Kinetic constants	Calculated parameters
*k*_*cat*_ (min^−1^)	6.71 ± 0.20
*k*_cat_/*K*_m_^acetyl-CoA^ (M^-1^ s^−1^)	4546
*k*_cat_/*K*_m_^4-MTOB^ (M^-1^ s^−1^)	2796

Reactions and global data fitting were performed as described in the Experimental procedures. Values are shown as average ± SD (*n* = 3).

### Reaction pH-dependence of BjMAMS2A

**Figure 3:**
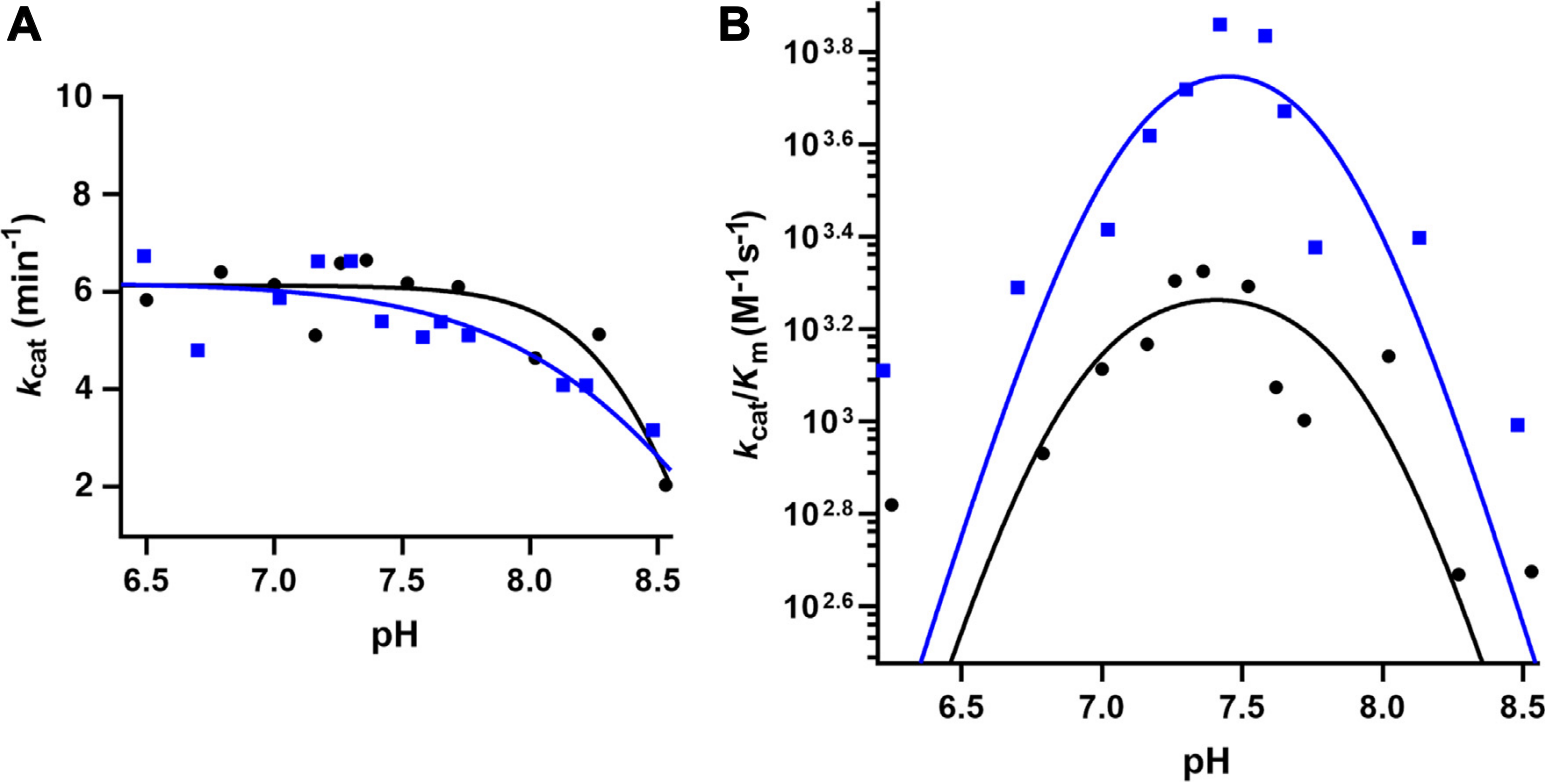
**pH-dependence of steady-state kinetic parameters of BjMAMS2A.***A*, plot of *k*_cat_*versus* pH with 4-MTOB (*circle*; *black*) and acetyl-CoA (*square*; *blue*) as varied substrates. *B*, plot of *k*_cat_/*K*_m_*versus* pH with 4-MTOB (*circle*; *black*) and acetyl-CoA (*square*; *blue*) as varied substrates. Experiments to determine kinetic parameters at each pH were performed in triplicate with each data shown the average value and with lines indicating the best of fit of the data, as described in Experimental procedures.

To evaluate the proposed role of general acid-base chemistry in the chemical mechanism of MAMS, the pH-dependence of *k*_cat_ and *k*_cat_/*K*_m_ for both 4-MTOB and acetyl-CoA was determined (Fig. 3). Comparison of *k*_cat_ over pH 6.5 to 8.5 for each substrate shows a decline in activity with increasing pH (Fig. 3*A*), which suggests that deprotonation of a residue (or residues) in the enzyme–substrate complex occurs. The 4-MTOB *k*_cat_ *versus* pH data displays a sharper decline (slope >1) and was best fit to the equation for two basic nonresolvable ionizable groups with p*K*_b_ = 8.45 ± 0.08. The acetyl-CoA *k*_cat_ *versus* pH data has a shallower decline (<1) and was best fit to the equation for one basic ionizable group with p*K*_b_ = 8.39 ± 0.16. The *k*_cat_/*K*_m_ pH profiles, which follow the ionization state of either free enzyme or free substrate, for 4-MTOB and acetyl-CoA were both bell-shaped with slopes greater than 1 on the acidic and basic sides (Fig. 3*B*). Fitting of the 4-MTOB *k*_cat_/*K*_m_ *versus* pH profile to the equation for two acidic nonresolvable and two basic nonresolvable ionizable groups estimates the p*K*_a_ values of the ionizable groups on the acidic and basic sides as p*K*_a_ = 6.85 ± 0.10 and p*K*_b_ = 7.96 ± 0.10, respectively. Fitting of the acetyl-CoA data to the same equation yields p*K*_a_ = 7.03 ± 0.20 and p*K*_b_ = 7.86 ± 0.22, respectively. Possible identities of the acidic and basic groups involved in the reaction mechanism are discussed below in relation to the characterization of site-directed mutants of BjMAMS2A.

The general acid residues observed in the *k*_cat_ pH profiles of BjMAMS2 are probably the same as those observed in the *k*_cat_/*K*_m_ pH profiles. The discrepancy of the p*K*_b_ values between the profiles may result from varying stickiness of the substrates in the BjMAMS2A active site, which can displace the p*K* in *k*_cat_/*K*_m_ profiles (32). This phenomenon was also observed in the pH-dependence of steady-state kinetic parameters of *Mycobacterium tuberculosis* IPMS (32). With the bacterial IPMS, the calculated p*K*_a_ and p*K*_b_ values for *k*_cat_/*K*_m_ with each substrate (KIV p*K*_a_ = 7.0 and p*K*_b_ = 8.5; acetyl-CoA p*K*_a_ = 6.5 and p*K*_b_ = 8.5) are like those of MAMS; however, IPMS displays an increase in *k*_cat_ as pH increases with its substrates (p*K*_a_ = 6.7) (30).

### Site-directed mutagenesis of BjMAMS2A active site residues

**Figure 4:**
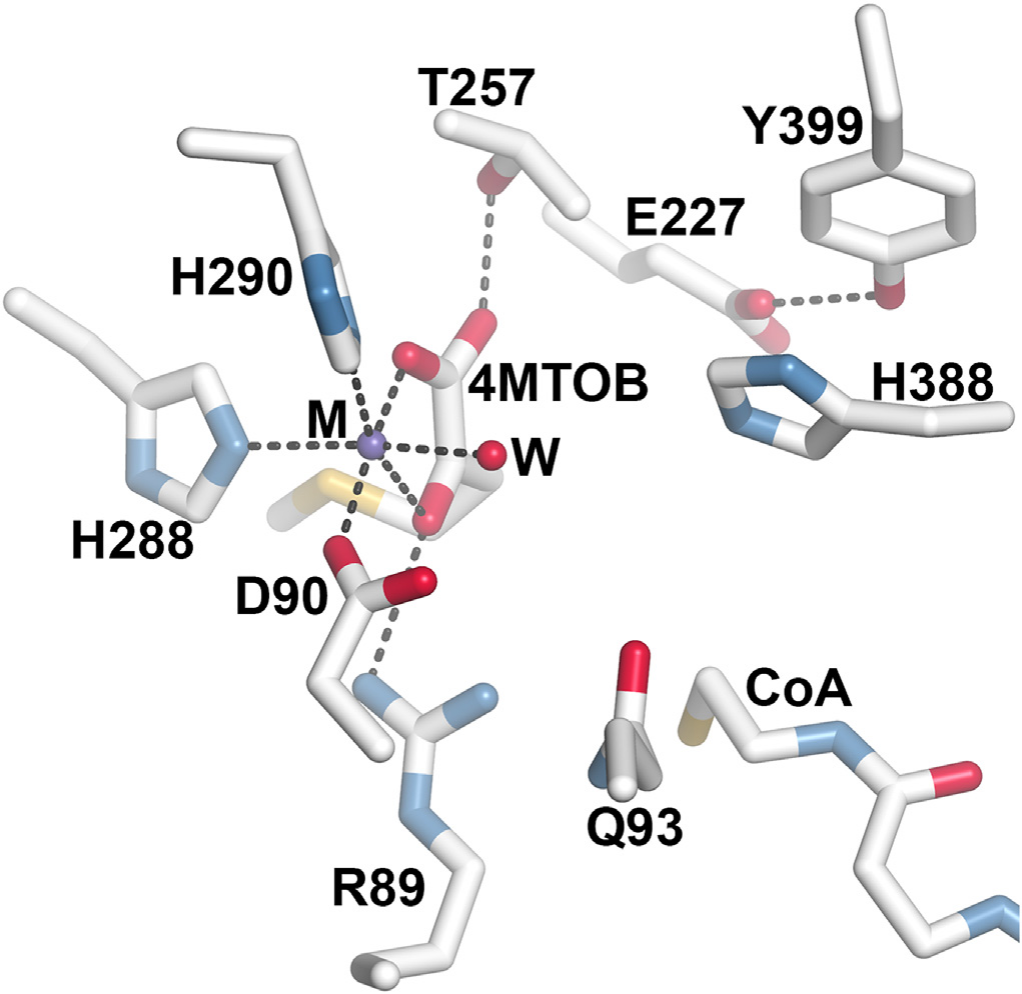
**Overview of the BjMAMS2A active site.** Residues in the active site of the x-ray crystal structure of the BjMAMS2•4–MTOB•CoA complex ((19); PDB: 6E1J) are shown as *stick* models with hydrogen bond/ionic interactions shown as *dotted* lines. M = Mn^2+^ and W = water.

To probe the chemical mechanism at the active site of BjMAMS2A, active site mutants were generated based on the x-ray crystal structure of the BjMAMS1A•CoA•4-MTOB dead-end complex (19). In the active site (Fig. 4), Asp90, His288, His290, a water, and 4-MTOB bind an essential metal ion (modeled as Mn^2+^ from crystallization and enzyme assay conditions). Arg89 and Thr257 form additional interactions with 4-MTOB. Gln93 and His388 are in bridging positions between 4-MTOB and the free thiol of bound CoA. His388 ring stacks with Tyr399, which forms a hydrogen bond with the side-chain of Glu227. Based on the structure, His388 was proposed to act as a general base during carbanion formation of the acetyl-CoA substrate in the condensation step and Arg89 as a potential general acid during nucleophilic attack of the carbanion on the C2-carbonyl of the 2-oxo acid substrate. A water activated by an unidentified general base in the active site may serve as a nucleophile that attacks the thioester carbonyl, which leads to the formation of tetrahedral intermediate that collapses to release CoA and the 2-malate–derived product. Here, the contributions of three centrally positioned active site residues (Arg89, Glu227, and His388), as well as residues involved in binding either 4-MTOB or acetyl-CoA (Thr257 and Gln93, respectively), were examined. Tyr399 was additionally investigated for its potential role in structuring the active site through interactions with His388 and Glu227.

**Figure 5:**
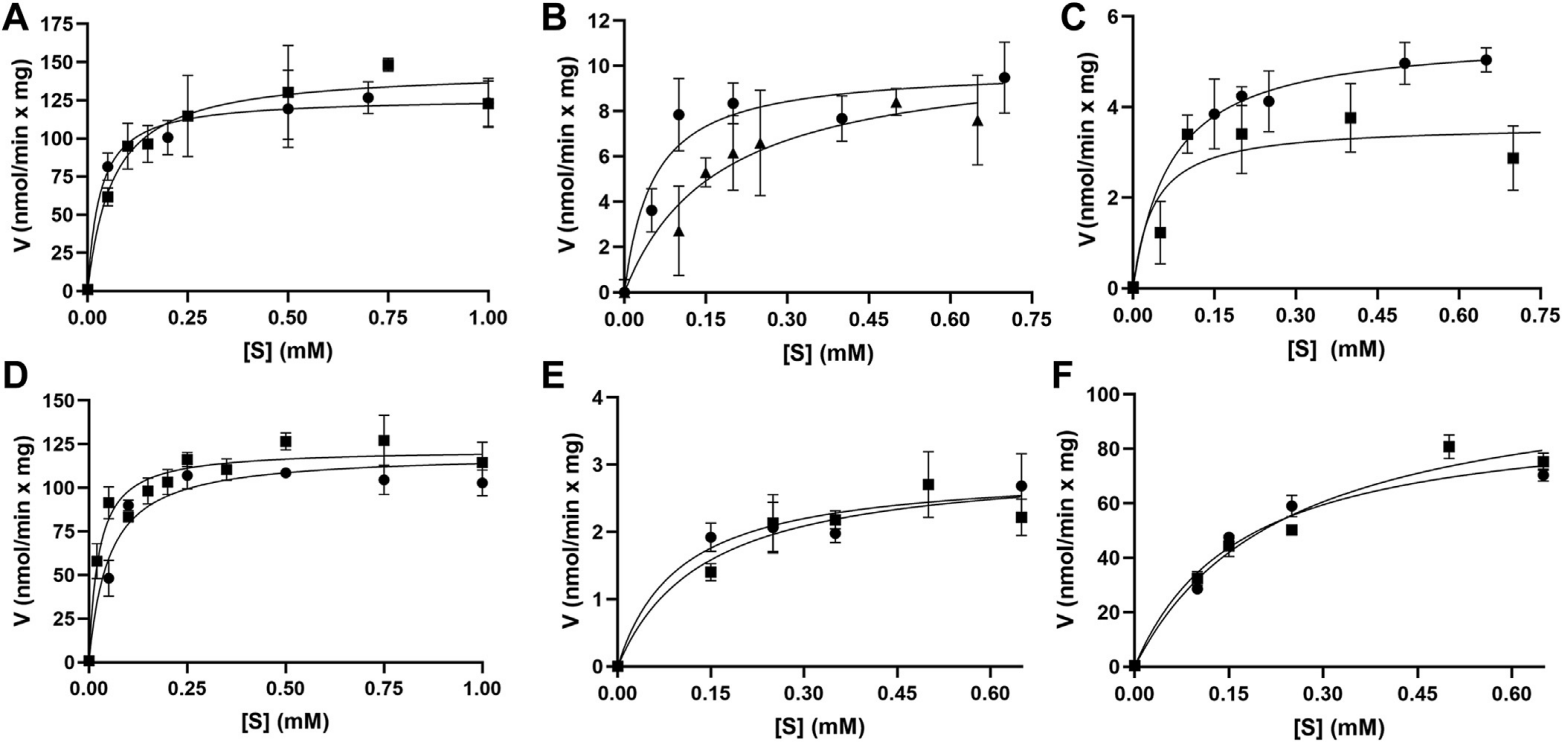
**Steady-state kinetic comparison of WT and mutant BjMAMS2A proteins.** Velocity *versus* substrate data for varied 4-MTOB (*circles*) and acetyl-CoA (*squares*) using (*A* and *D*) WT, (*B*) E227Q, (*C*) H388N, (*E*) Y399F, and (*F*) T257S BjMAMS2A are shown as average ± SD (n = 3). Panels *A* and *D* show data obtained with Mn^2+^ and Mg^2+^ as metal cofactors, respectively. Data were fit to the Michaelis–Menten equation as described in the Experimental procedures.

A series of point mutants targeting Arg89 (R89A, R89K, R89Q), Glu227 (E227A, E227D, E227Q), His388 (H388A, H388N, H388Q, H388D, H388E), Thr257 (T257A, T257S), Gln93 (Q93A, Q93N), and Tyr399 (Y399S, Y399F) of BjMAMS2A were generated as described in the Experimental procedures. Each mutant protein was expressed and purified from *E. coli* to levels comparable with WT enzyme (Fig. S2). Initial activity assays (using up to 100 μg protein) showed that only four mutants—E227Q, H388N, T257S, and Y399F—retained enzymatic activity. Steady-state kinetic analysis of WT, E227Q, H388N, T257S, and Y399F BjMAMS2A proteins was performed (Fig. 5; Table 2). The E227Q, H388N, and Y399F point mutations primarily impact turnover rate (*k*_cat_) with 13- to 14-fold decreases for both substrates observed with the E227Q mutant, 25- to 35-fold decreases for both substrates for the H388N mutant, and 39- to 41-fold decreases for both substrates for the Y399F mutant (Table 2). There are less than 3-fold changes in *K*_m_ compared to WT for the E227Q and H388N mutants and 2- to 5-fold changes for the Y399F mutant (Table 2). The T257S point mutation primarily affects *K*_m_ with a 3- to 9-fold increases for both substrates compared to WT (Table 2). The *k*_cat_ of the T257S mutant is comparable to that of WT with less than a 2-fold decrease for both substrates (Table 2). Below we discuss the potential roles of the residues mutated in this study.

**Table 2:** Steady-state kinetic analysis of WT and mutant BjMAMS2A

Protein	Varied substrate	*k*_cat_ (min^−1^)	*K*_m_ (μM)	*k*_cat_/*K*_m_ (M-^1^ s^−1^)
WT	acetyl-CoA	7.98 ± 0.42	63.4 ± 15	2098
WT	4-MTOB	6.97 ± 0.30	31.1 ± 9	3735
E227Q	acetyl-CoA	0.58 ± 0.09	174 ± 70	56
E227Q	4-MTOB	0.55 ± 0.04	53.0 ± 19	173
H388N	acetyl-CoA	0.31 ± 0.02	70.4 ± 21	73
H388N	4-MTOB	0.20 ± 0.02	37.9 ± 20	88
WT∗	acetyl-CoA	6.72 ± 0.17	24.6 ± 4	4553
WT∗	4-MTOB	6.58 ± 0.18	51.1 ± 10	2146
T257S∗	acetyl-CoA	5.98 ± 0.39	238 ± 38	419
T257S∗	4-MTOB	5.12 ± 0.20	169 ± 20	505
Y399F∗	acetyl-CoA	0.17 ± 0.02	140 ± 65	20
Y399F∗	4-MTOB	0.16 ± 0.01	99.6 ± 40	27

Initial velocity assays were performed as described in the Experimental procedures. Values are shown as average ± SD (*n* = 3). Enzyme activities for proteins marked with (∗) were determined using Mg^2+^ as a metal cofactor, as opposed to Mn^2+^.

In the MAMS active site, Arg89 interacts with the carbonyl of 4-MTOB through a guanidinium group nitrogen with another nitrogen oriented toward the space bridging 4-MTOB and CoA (Fig. 4). Substitution of this residue with alanine, lysine, or glutamine results in a loss of enzymatic activity, which confirms the importance of having the guanidinium group in the active site. Across other sub-groups (*i.e.*, carboxylase-, aldolase-, and lyase-like) of the DRE-TIM metallolyase superfamily, similar losses of activity have been reported for mutations of this structurally conserved arginine (27, 33, 34, 35). Based on the pH-dependence data (Fig. 3), Arg89 is unlikely to serve as the general acid in the MAMS reaction as originally proposed (19). Even with a perturbed value in the active site, the p*K*_a_ (∼12.5) would be higher than the MAMS optimum pH of 7.5. In malate synthase, another member of the Claisen-condensation branch of the DRE-TIM metallolyase superfamily (28, 36), the analogous arginine plays a similar stabilizing role in the reaction with side-chain interactions bridging the oxo acid substrate and enolate intermediate. Moreover, computational studies on the citrate synthase, another DRE-TIM metallolyase family member, suggest that the preferred energetic pathway to product formation does not use an arginine as a general acid (37). Thus, the p*K*_b_ values (8.39–8.45) determined from the *k*_cat_ *versus* pH profiles of BjMAMS2A (Fig. 3*A*) may point to the functional significance of Arg89 in substrate binding and stabilizing the enolate intermediate of the MAMS reaction, instead of a direct catalytic role.

Glu227 is positioned further away from the catalytic center of MAMS and forms an interaction from its carboxylate side-chain to the hydroxyl group of Tyr399, which in turn ring-stacks with His388 (Fig. 4). Of the three mutations at this position, only the isosteric change of E227Q retains enzymatic activity with 37- and 22-fold reductions in catalytic efficiency (*k*_cat_/*K*_m_) for acetyl-CoA and 4-MTOB, respectively (Table 2). The E227A and E227D mutants had no detectable activity. This result indicates that the glutamate carboxylate is important, but not necessary for catalysis. It is possible that the alanine and aspartate mutations break interaction with Tyr399, which may alter its positioning, as well as that of His388. For example, perturbation of the active site around the catalytic histidine in citrate synthase negatively impacts transition state interactions and kinetic parameters (38, 39). Moreover, although Glu227 does not directly interact with His388 and is unlikely to act as a reactive group in the chemical mechanism, its proximity to His388 and/or its role in positioning Tyr399 for stacking with His388 may enhance the basicity of the histidine (40, 41).

Of the residues targeted for site-directed mutagenesis, His388 is the mostly likely general base in the MAMS reaction. The p*K*_a_ values of 6.83 to 7.04 determined for BjMAMS2A pH-dependence analyses are consistent with the involvement of a histidine in the reaction mechanism. Either removal of the imidazole side-chain (H388A) or substitution with acidic groups (H388D and H388E) yields no detectable activity. The H388N and H388Q mutants were made to probe the contribution of the N_δ_ and N_ε_, respectively, of the imidazole side-chain and to remove the ability to act as a general base, while retaining hydrogen bonding capacity. Only the H388N mutant had detectable activity but with 29- and 42-fold lower *k*_cat_/*K*_m_ values for acetyl-CoA and 4-MTOB, respectively (Table 2). Structurally, the position of His388 in MAMS is analogous to the catalytic histidine of citrate synthase, which facilitates the Claisen condensation of oxaloacetate and acetyl-CoA (37, 38, 39).

In addition to His388's role as a general base, in the deprotonated form, histidine can form π-π stacking interactions with adjacent aromatic residues (42). In the x-ray crystal structure of BjMAMS1A (19), the aromatic ring of Tyr399 is parallel to and 4 Å from the imidazole ring of His388. Interestingly, the sequence and structural arrangement of Glu227, Tyr399, and His388 in MAMS is conserved in IPMS (Fig. S3) (31). Ring stacking interactions in the active sites of glutathione-S-transferases and ATPases can influence enzyme efficiency (40, 41). It is possible that during formation of the enolate intermediate, the His388–Tyr399 interaction may help stabilize this reaction intermediate. The effect of Tyr399 point mutations are consistent with a role for ring stacking interactions at this position for efficient catalysis, as substitution of Tyr399 with serine results in the loss of enzyme activity, while substitution with phenylalanine yields detectable activity, but with very low *k*_cat_/*K*_m_ values for acetyl-CoA and 4-MTOB (Table 2). The decrease in activity with the phenylalanine substitution in comparison to WT points to the importance of the hydroxyl group of Tyr399 for interaction with Glu227 and its positioning in the MAMS active site, as discussed previously.

The positions of Thr257 and Gln93 in the x-ray crystal structure of BjMAMS1A (19), with the hydroxyl group of Thr257 hydrogen-bonding to the C1-carbonyl group of 4-MTOB and the amide group of Gln93 being in proximity to the free sulfhydryl group of CoA suggests that these residues are involved in substrate binding (Fig. 4). Substitution of both residues with alanine in BjMAMS2A results in a loss of enzyme activity, indicating the importance of Thr257 and Gln93 to the active site. Substitution of Thr257 with serine yields a level of enzyme activity comparable to WT with less than a 2-fold decrease in *k*_cat_; however, there is a 4- to 10-fold decrease in *k*_cat_/*K*_m_ for both substrates due to 3- to 9-fold increases in their *K*_m_ values (Table 2). These results indicate that disruption of substrate binding at the active site of the mutant due to the serine substitution, supporting the primary role of Thr257 in substrate binding. Substitution of Gln93 with asparagine yields no detectable activity, indicating that the positioning of the amide group with respect to the acetyl-CoA substrate may help guide this substrate interaction. Apart from substrate binding, positioning of Gln93 may also have a role in intermediate stabilization due to its proximity to Arg89, which is proposed to play a role in stabilizing the enolate intermediate, as discussed previously.

### Revised MAMS chemical mechanism

**Figure 6:**
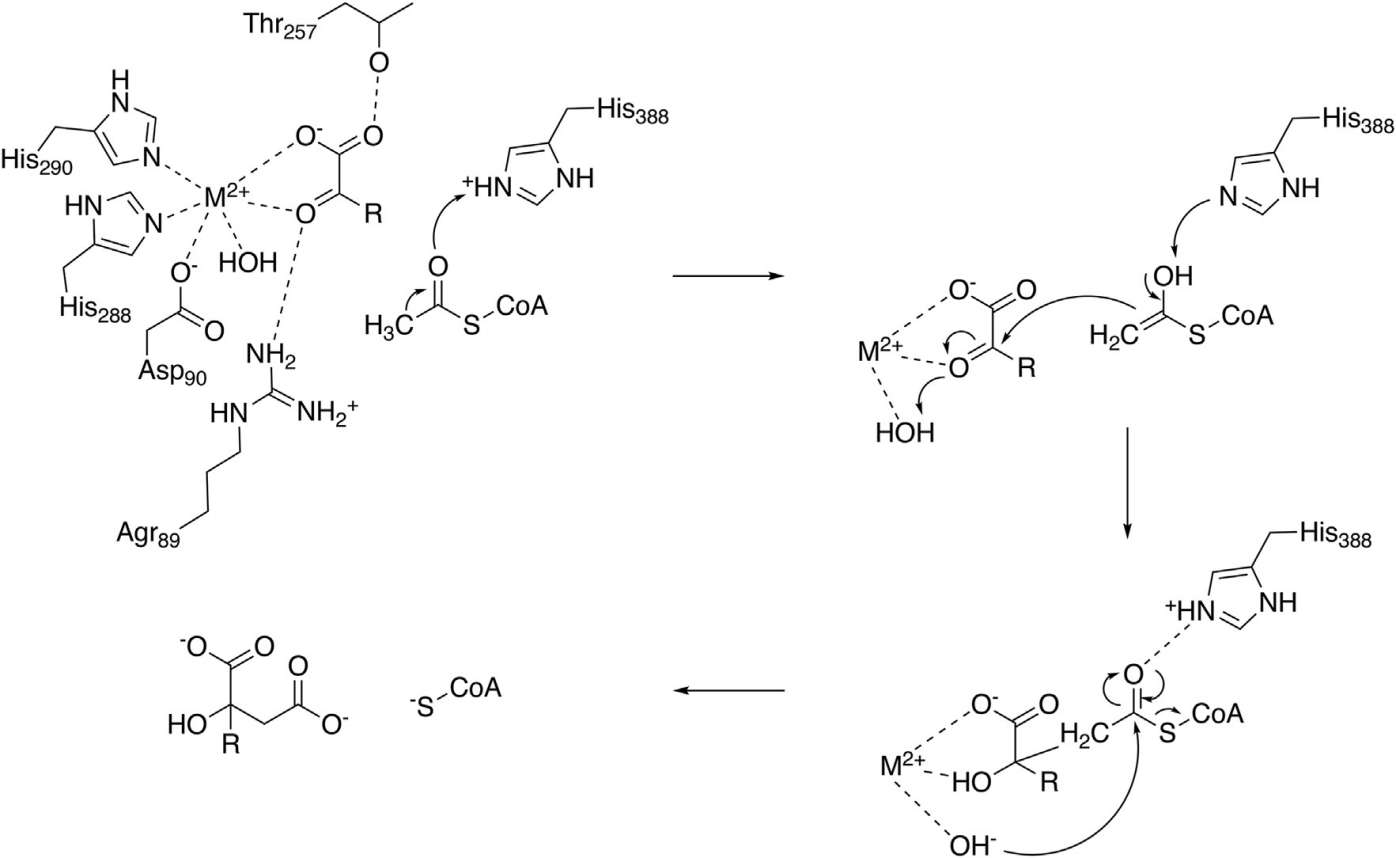
**Revised chemical mechanism for MAMS**.

Based on previous structural information (19) and the biochemical data presented here, an updated reaction mechanism for the MAMS-catalyzed Claisen condensation of 4-MTOB and acetyl-CoA is proposed (Fig. 6). The crystal structure of MAMS complexed with 4-MTOB, CoA, and metal ion (Mn^2+^) helps define critical features of the active site, especially the binding site for the required divalent metal. Based on the structure, the metal is bound by two uncharged histidines (His288 and His290), the carboxylate of Asp90, two oxygens of the substrate (drawn as the 2-carbonyl and terminal carboxylate), and a water molecule. The 4-MTOB substrate is also held in position by interactions with Arg89 and Thr257 and blocked by the binding of acetyl-CoA, which is consistent with biochemical data indicating an ordered kinetic mechanism (Fig. 2; Table 1). The pH-dependence of *k*_cat_/*K*_m_ (Fig. 3B), which corresponds to free enzyme or free substrate, suggests that titration of two residues with increasing pH leads to optimal activity; this may reflect titration of the metal-binding histidines. The decrease in *k*_cat_/*K*_m_ at more basic pH may reflect acetyl-CoA binding, as there are four arginines and a lysine that interact with this substrate (19). Overall, the MAMS active site is highly structured around the metal site for substrate binding and catalysis; this is a central feature across the DRE-TIM metallolyase superfamily (27, 28).

Within the MAMS active site, His388 is ideally positioned as the key catalytic residue for enolization and condensation steps in the reaction sequence (Figures 4 and 6). Although a structure of MAMS (or IPMS) with acetyl-CoA remains to be determined, the position of CoA in the MAMS active site suggests that Arg89 and Gln83 likely help position the acetyl-group for the reaction. As described above, the arginine is invariant across DRE-TIM metallolyases (27, 33, 34, 35) and essential for enzymatic activity. Moreover, comparison of the MAMS (Fig. 4) and IPMS (Fig. S3*A*) active sites suggests that positioning of the glutamine side-chain may change orientation between the apoenzyme (pointing away from the active site, as observed in IPMS) and CoA-bound form (pointing into the active site, as observed in MAMS). Binding of acetyl-CoA into the MAMS active site relative to His388 would readily facilitate the enolization step in the reaction sequence as a general acid and then serve as a general base in the condensation step (Fig. 6). It should be noted that the exact mechanism of enolization in DRE-TIM metallolyases varies. For example, in citrate synthase, it is concerted (43), whereas malate synthase uses a stepwise mechanism (44). Studies of the bacterial IPMS showed an absence of primary deuterium isotope effects, which prevented similar analysis of enolization in the closest relative of MAMS (30).

Following enolization (Fig. 6), the condensation step can readily occur with His388 serving as a general base. In malate and citrate synthases, acidic residues are positioned to serve as general acids; however, MAMS lacks a comparably positioned residue. Asp90 is unlikely to serve this role, as the interaction with metal would maintain the side-chain carboxylate; however, the water molecule that interacts with the bound metal in the MAMS active site is proximal to the substrate carbonyl attacked by the enolate. The resulting alkoxide, stabilized by the metal ion, would be available for nucleophilic attack on the thioester carbonyl and, after subsequent collapse of the tetrahedral intermediate, release free CoA and the extended 2-malate derivative (Fig. 6). Moreover, given the structural and sequence conservation of the MAMS and IPMS active sites (Fig. S3*B*), the leucine biosynthesis enzyme likely employs a similar catalytic mechanism.

In summary, this work provides more insights into the structure and biochemistry of MAMS, the key enzyme in the glucosinolate biosynthetic pathway in Brassica plants. These insights could be a useful addition to the breadth of knowledge required for efforts in metabolically engineering glucosinolates in plants and bacteria for plant defense, human health, and nutrition.

## Experimental procedures

### Protein expression and purification

The pET-28a-BjMAMS2A expression construct was previously described (19). For recombinant protein expression, the expression vector was transformed into *E. coli* Rosetta II (DE3) cells. Cells were grown in Terrific broth with 50 μg mL^−1^ kanamycin until A_600nm_ ∼0.6, after which protein expression was induced by the addition of IPTG (1 mM final) and grown overnight at 16 °C. After centrifugation (10,000*g*; 10 min), the cell pellet was dissolved in lysis buffer (50 mM Tris–HCl, pH 7.5, 20 mM imidazole, 500 mM NaCl, 1% (v/v) Tween-20, and 10% (v/v) glycerol) with cell lysis by sonication at 4 °C. After clarification (30,000*g*; 60 min), the supernatant was passed over an Ni^2+^-NTA-agarose column at room temperature. The column was washed with lysis buffer lacking Tween-20 and bound protein was eluted in elution buffer (50 mM Tris–HCl, pH 7.5, 250 mM imidazole, 500 mM NaCl, and 10% (v/v) glycerol). Eluted protein was further purified by size-exclusion chromatography using an Akta FPLC with a Sephadex-200 column equilibrated with 25 mM N-[2-hydroxyethyl]-piperazine-N′-[2-ethanesulfonic acid] (Hepes), pH 7.5, 100 mM NaCl, and 1 mM DTT. All buffers were chilled at 4 °C before use. Protein concentration was determined using the Bradford method with bovine serum albumin as the standard.

### Enzyme assays for steady-state kinetics

Initial velocity data for the condensation reaction catalyzed by BjMAMS2A were determined using 4,4′-dithiodipyridine to detect the formation of free CoA at A_324 nm_ (ε = 19,800 M^−1^ cm^−1^) at 25 °C (45). A typical reaction mix contained 50 mM Hepes at pH 7.5, 20 mM KCl, 20 mM MgCl_2_, 200 μM 4,4′-dithiodipyridine, 0.5 mM acetyl-CoA, and 1 mM 4-MTOB. Reactions were initiated by the addition of the enzyme, typically at a 1.8 μM final concentration. Initial velocity assays of BjMAMS2A used either a range of 0.02 to 1 mM acetyl-CoA with 1 mM 4-MTOB or a range of 0.05 to 1 mM 4-MTOB with 0.5 mM acetyl-CoA. All assays were performed in triplicate. Steady-state kinetic parameters (*V*_max_ and *K*_m_) were determined by fitting initial velocity data to the Michaelis–Menten equation using GraphPad Prism. For analysis of the steady-state kinetic mechanism, initial velocity rates were measured as above with a matrix of 4-MTOB and acetyl-CoA concentrations (each data point in triplicate) with resulting data fit to rate equations describing ordered sequential, *v* = (*V*_max_ [A] [B])/(*K*_A_ *K*_B_ + *K*_B_ [A] + [A] [B]), and random sequential, v = (*V*_max_ [A] [B])/(α *k*_A_ *k*_B_ + *K*_B_ [A] + *K*_A_ [B] + [A] [B]), kinetic mechanisms, where *v* is the initial velocity, *V*_max_ is the maximum velocity, *K*_A_ and *K*_B_ are the *K*_M_ values for substrates A and B, respectively, and α is the interaction factor if the binding of one substrate changes the dissociation constant for the other substrate (29). SigmaPlot was used for global fitting analysis.

### Dependence of steady-state kinetics by pH

The pH-dependence of *k*_cat_ and *k*_cat_/*K*_m_ with 4-MTOB was determined by varying the concentration of 4-MTOB (0.05–1 mM) at 0.5 mM acetyl-CoA and varying the concentration of acetyl-CoA (0.02–1 mM) at 1 mM 4-MTOB. Experiments were performed in triplicate. A mixed buffer system (50 mM acetic acid/50 mM 2-(N-morpholino)ethanesulfonic acid/100 mM Tris; (46)) was used in place of Hepes buffer to maintain constant ionic strength across the experimental pH range (pH 6.5–8.5). The dependence of kinetic parameters *versus* pH profile data were fitted to the equations for two acidic nonresolvable and two basic nonresolvable ionizable groups (*y* = *C*/[1 + (*H*^2^/*K*_a_^2^) + (*K*_b_^2^/*H*^2^)]), two basic nonresolvable ionizable groups (*y* = *C*/[1 + (*K*_b_^2^/*H*^2^)]), or one basic ionizable group (*y* = *C*/[1 + *K*_b_/*H*]) using GraphPad Prism with *C* fixed at the pH-independent plateau value; *H* is the hydrogen ion concentration, and *K*_a_ and *K*_b_ are the acidic and basic p*K*_a_ constants for the ionizable groups, respectively.

### Site-directed mutagenesis and mutant protein analysis

To construct BjMAMS2A mutants, the pET-28a-BjMAMS2A vector was used as a template in QuikChange site-directed mutagenesis method (Agilent Technologies) with mutant oligonucleotides (Table S1) to generate the following point mutants: R89A, R89K, R89Q, E227A, E227D, E227Q, H388A, H388N, H388Q, H388D, and H388E. The genes for the following point mutants were synthesized and subcloned into the pET-28a vector by GenScript: Q93A, Q93N, T257A, T257S, Y399S, and Y399F. Each resulting pET-28a-BjMAMS2A mutant plasmid was confirmed by DNA sequencing and transformed into *E. coli* Rosetta II (DE3) for protein expression, purification, and enzymatic activity analysis, as described above. Enzymatic activities of the H388N and E227Q mutants, as well as WT MAMS2A, were analyzed using 10 mM MnCl_2_ instead of 20 mM MgCl_2_ as the metal cofactor after a higher activity was observed for the mutants in the presence of Mn^2+^.

## Data availability

All data are contained within the article.

## Supporting information

This article contains supporting information.

## Conflicts of interest

J. M. J. is an associate editor of this journal. The other author declares that they have no conflicts of interest with the contents of this article.
