# Supplementary material for: Kinetic and catalytic mechanisms of the methionine-derived glucosinolate biosynthesis enzyme methylthioalkylmalate synthase
Source: J Biol Chem. 2024 Sep 23;300(11):107814. doi: 10.1016/j.jbc.2024.107814 (PMC11532901; doi:10.1016/j.jbc.2024.107814)

**SUPPORTING INFORMATION**

**Kinetic and Catalytic Mechanism of the Methionine-Derived Glucosinolate Biosynthesis Enzyme Methylthioalkylmalate Synthase**

*Vivian Kitainda^a^, Joseph M. Jez^a,*^*

Includes Supporting Table S1, Figure S1, Figure S2, and Figure S3.

**Supplemental Table S1. BjMAMS2A site-directed mutagenesis oligonucleotide pairs.** Mutated codon positions are shown in bold.

R89A_Forward 5'-dGTATTCGACACGACGCT**CGC**TGACGGTGAACAAGCTC-3'
R89A_Reverse: 5'-dGAGCTTGTTCACCGTCA**GCG**AGCGTCGTGTCGAATAC-3'

R89K_Forward 5'-dGTATTCGACACGACGCTC**AAA**GACGGTGAACAAGCTCC-3'
R89K_Reverse 5'-dGGAGCTTGTTCACCGTC**TTT**GAGCGTCGTGTCGAATAC-3'

R89Q_Forward 5'-dCGACACGACGCTC**CAA**GACGGTGAACAAG-3'
R89Q_Reverse 5'-dCTTGTTCACCGTC**TTG**GAGCGTCGTGTCG-3'

E227A_Forward 5'-dCAACTTGGTTGCG**CG**GATGGCGGCAGG-3'
E227A_Reverse 5'-dCCTGCCGCCATC**CG**CGCAACCAAGTTG-3'

E227D_Forward 5'-dCAACTTGGTTGCGA**T**GATGGCGGCAGGTC-3'
E227D_Reverse 5'-dGACCTGCCGCCATC**A**TCGCAACCAAGTTG-3'

E227Q_Forward 5'-dCAACTTGGTTGC**C**AAGATGGCGGCAG-3'
E227Q_Reverse 5'-dCTGCCGCCATCTT**G**GCAACCAAGTTG3'

H388A_Forward 5'-dCATGAGAGCGGCATT**GCC**CAGGATGGAATCTTG-3'
H388A_Reverse 5'-dCAAGATTCCATCCTG**GGC**AATGCCGCTCTCATG-3'

H388N_Forward 5'-dCATGAGAGCGGCATT**AAC**CAGGATGGAATC-3'
H388N_Reverse 5'-dGATTCCATCCTG**GTT**AATGCCGCTCTCATG-3'

H388Q_Forward 5'-dGAGAGCGGCATT**CAA**CAGGATGGAATCTTG-3'
H388Q_Reverse 5'-dCAAGATTCCATCCTG**TTG**AATGCCGCTCTC-3'

H388D_Forward 5'-dCATGAGAGCGGCATT**G**ACCAGGATGGAATC-3'
H388D_Reverse 5'-dGATTCCATCCTGGT**C**AATGCCGCTCTCATG-3'

H388E_Forward 5'-dCATGAGAGCGGCATT**GAA**CAGGATGGAATCTTG-3'
H388E_Reverse 5'-dCAAGATTCCATCCTG**TTC**AATGCCGCTCTCATG-3'

**Supplemental Figure S1**. **Secondary plot of double-reciprocal (or Lineweaver-Burk; LB) plot slopes versus 1 / [fixed substrate] for BjMAMS2A**. Slopes for varied acetyl-CoA (open circles; fixed 4-MTOB) and varied 4-MTOB (filled circles; fixed acetyl-CoA) are shown.


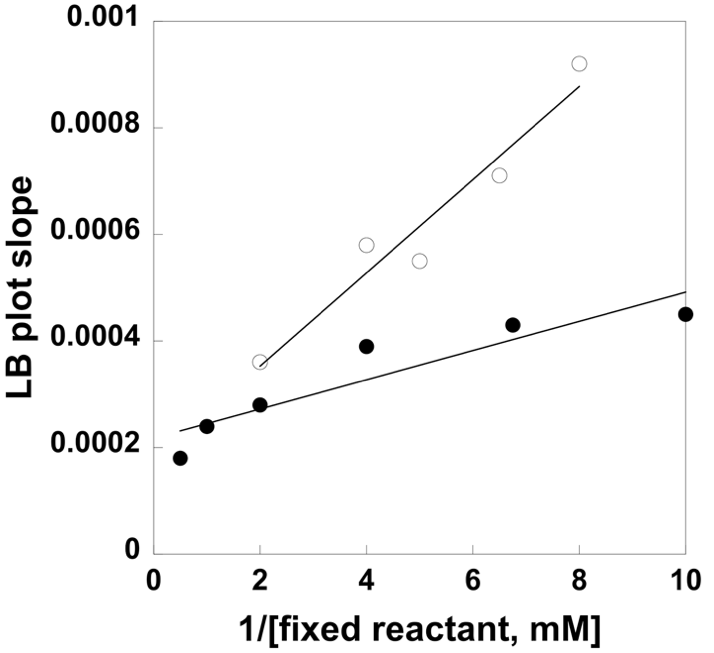


**Supplemental Figure S2**. **SDS-PAGE analysis of purified wild-type and mutant BjMAMS2A proteins.** Each protein was expressed and purified as described in the Experimental Procedures. Samples (~5 μg per lane) were analyzed by SDS-PAGE and stained for total protein using Coomassie Blue. Selected molecular weights in kDa for the protein ladder are indicated (left) with wild-type and mutant BjMAMS2A form noted above each lane.

**35**

**50**

**70**

**100**


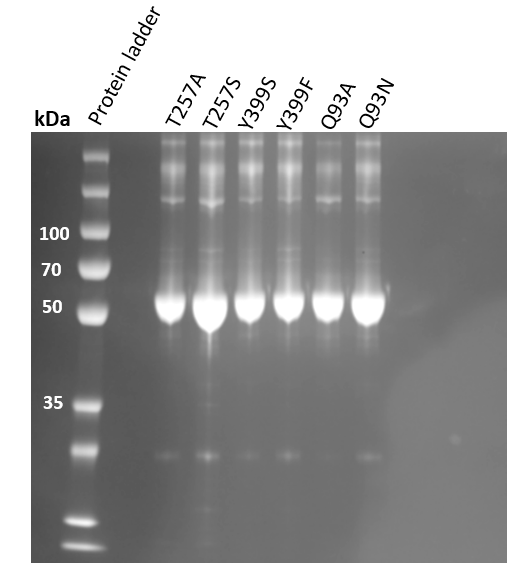

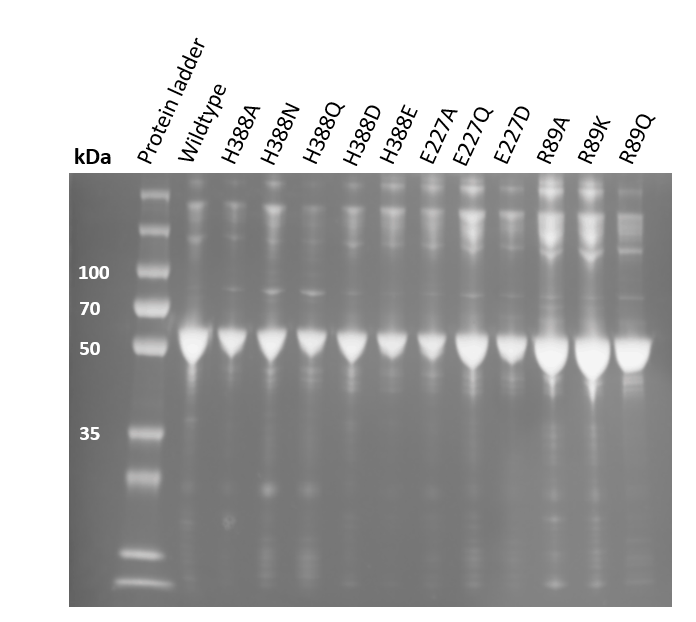


**Supplemental Figure S3**. **Overview of the bacterial IPSM active site and MAMS and IPMS active site sequences**. (**a**) Residues in the active site of the x-ray crystal structure of the *M. tuberculosis* IPMS in complex with KIV (ref 32; PDB: 1SR9) are shown as stick models with hydrogen bond/ionic interactions shown as dotted lines and the metal ion (modeled as Zn^2+^ in the structure) as a sphere. The view is from a similar orientation as the BjMAMS active site shown in Figure 4. (**b**) Targeted sequence alignment of MAMS isoforms from *B. juncea* (BjMAMS2A/C5J4P4, BjMAMS2B/C5J4N9, BjMAMS1A/C5J4P1, BjMAMS1B/C5J4N7) and *A. thaliana* (AtMAMS1/Q9FG67, AtMAMS2/Q8VX04; AtMAMS3/Q9FN52), as well as IPMS isoforms from *A. thaliana* (AtIPMS1/A0A178WDC7; AtIPMS2/Q9C550), *E. coli* (EcIPMS/P09151), and *M. tuberculosis* (MtIPMS; P9WQB3). UniProt codes are provided after each abbreviation. Residue numbering for BjMAMS2A and MtIPMS active site residues are shown at the top and bottom, respectively.


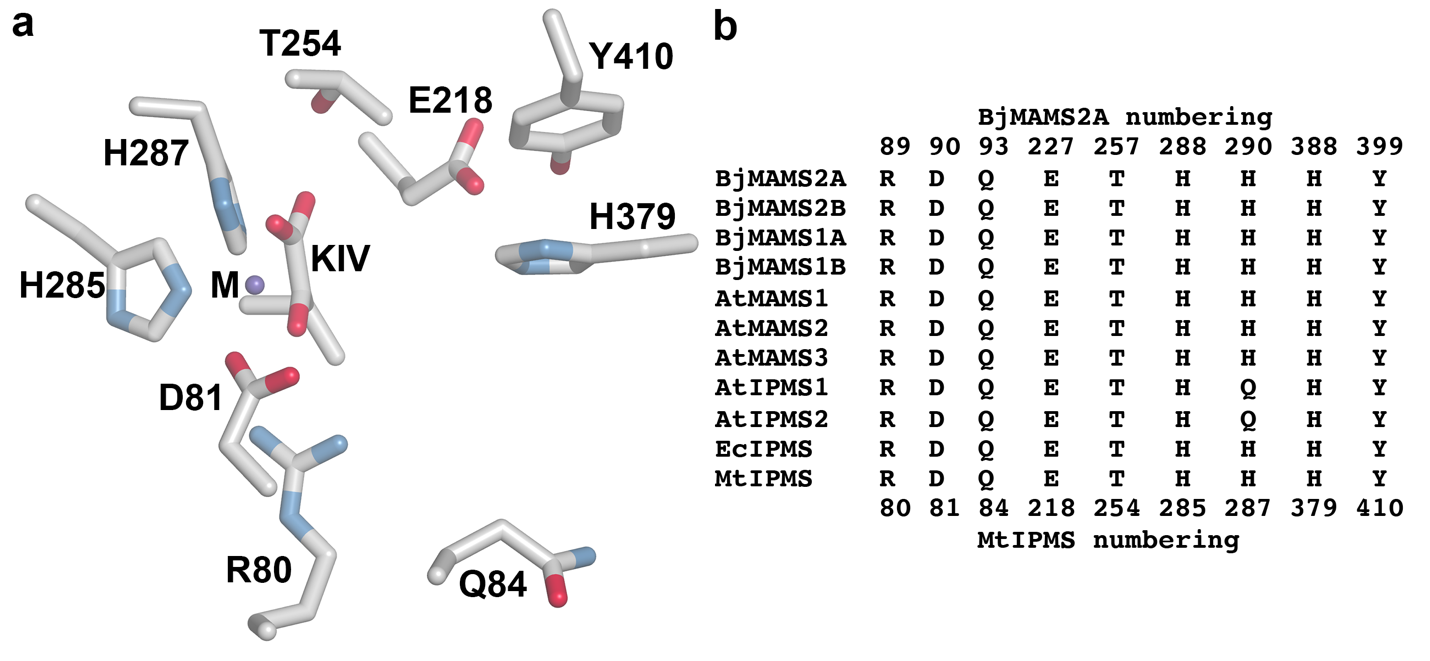

Supplement: Supplemental Table S1, and Figures S1–S3 [file mmc1.docx]
